# Electron-Impact Total Ionization Cross Sections of Hydrocarbon Ions

**DOI:** 10.6028/jres.107.007

**Published:** 2002-02-01

**Authors:** Karl K. Irikura, Yong-Ki Kim, M. A. Ali

**Affiliations:** National Institute of Standards and Technology, Gaithersburg, MD 20899-0001; Department of Chemistry, Howard University, Washington, DC 20059

**Keywords:** CH_2_^+^, CH_3_^+^, CH_4_^+^, C_2_H_2_^+^, C_2_H_4_^+^, C_2_H_6_^+^, H_3_O^+^, electron-impact ionization, molecular ions

## Abstract

The Binary-Encounter-Bethe (BEB) model for electron-impact total ionization cross sections has been applied to ${\mathrm{CH}}_{2}^{+}$, ${\mathrm{CH}}_{3}^{+}$, ${\mathrm{CH}}_{4}^{+}$, $C_{2}H_{2}^{+}$, $C_{2}H_{4}^{+}$, $C_{2}H_{6}^{+}$ and H_3_O^+^. The cross sections for the hydrocarbon ions are needed for modeling cool plasmas in fusion devices. No experimental data are available for direct comparison. Molecular constants to generate total ionization cross sections at arbitrary incident electron energies using the BEB formula are presented. A recent experimental result on the ionization of H_3_O^+^ is found to be almost 1/20 of the present theory at the cross section peak.

## 1. Introduction

Ionization cross sections for atomic and molecular ions are among the critical data needed in modeling plasmas in fusion devices. Hydrocarbon molecules and their ion fragments are formed inside a tokamak in edge plasmas and near a divertor. The Binary-Encounter-Bethe (BEB) model [1] has successfully generated reliable total ionization cross sections of small as well as large molecules [2–6]. The BEB model combines a modified form of the Mott cross section with the asymptotic form of the Bethe theory (i.e., high incident energy *T*) for electron-impact ionization of a neutral atom or molecule. The original BEB model was slightly modified for applications to atomic and molecular ions [7].

In this article we apply the modified BEB formula for ions to hydrocarbon ions of interest to magnetic fusion: ${\mathrm{CH}}_{2}^{+}$, ${\mathrm{CH}}_{3}^{+}$, ${\mathrm{CH}}_{4}^{+}$, $C_{2}H_{2}^{+}$, $C_{2}H_{4}^{+}$, $C_{2}H_{6}^{+}$, and H_3_O^+^. We outline the theory in Sec. 2, and our theoretical results are presented in Sec. 3. A recent experiment on the formation of H_3_O^++^ by electron impact [8] is compared to the present theory in Sec. 3.

## 2. Outline of Theory

The BEB formula for ionizing an electron from a molecular orbital of a neutral molecule by electron impact is [1]:

$$\sigma_{\mathrm{BEB}}=\frac{S}{t+u+1}\left[\frac{\ln\;t}{2}\left(1-\frac{1}{t^{2}}\right)+1-\frac{1}{t}-\frac{\ln\;t}{t+1}\right],$$ (1)

where *t* = *T*/*B*, *u* = *U*/*B*, $S=4\pi a_{0}^{2}N\;R^{2}/B^{2}$, *a*_0_ is the Bohr radius (= 0.5292 Å), *R* is the Rydberg energy (= 13.6057 eV), *T* is the incident electron energy, and *N*, *B*, and *U* are the electron occupation number, the binding energy, and the average kinetic energy of the orbital, respectively.

In Eq. (1), the terms in the square brackets are based on the Mott theory and the Bethe theory. However, the denominator *t* + *u* + 1 is based on a plausible, but less rigorous argument, i.e., the effective kinetic energy of the incident electron seen by the bound target electron should be the incident electron energy *T* plus the potential energy *U* + *B* of the target electron [9]. Hence the *T* in the denominator of the original Mott and Bethe theories was replaced by *T* + *U* + *B*, or *t* + *u* + 1 in Eq. (1), where *B* is used as the energy unit.

The net effect of using *t* + *u* + 1 instead of *t* in the denominator of Eq. (1) is to reduce substantially the cross section near the ionization threshold. This modification was found not only to be effective but also absolutely necessary to have the theory agree with reliable experimental ionization cross sections near the threshold for many neutral atoms and molecules.

In a previous article [7] for singly charged molecular ions, we have shown that the denominator *t* + *u* + 1 is replaced by *t* + (*u* + 1)/2 to generate ionization cross sections in good agreement with available experimental data. The modified BEB equation for singly charged ions is:

$$\sigma_{\mathrm{ion}}=\frac{S}{t+\left(u+1\right)/2}\left[\frac{\ln\;t}{2}\left(1-\frac{1}{t^{2}}\right)+1-\frac{1}{t}-\frac{\ln\;t}{t+1}\right].$$ (2)

Equation (2) is as simple as the BEB formula for neutral targets, Eq. (1), and does not require any more input data than the original BEB formula.

## 3. Theoretical Results

**Fig. 1:**
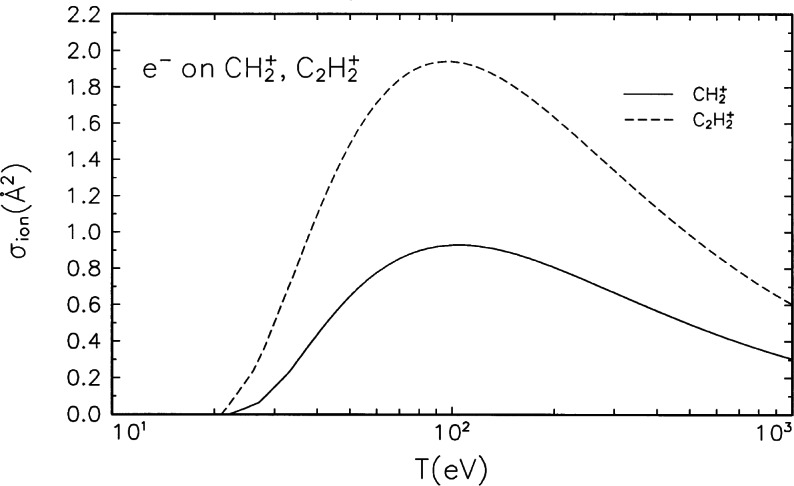
Electron-impact ionization cross sections for ${\mathrm{CH}}_{2}^{+}$ and $C_{2}H_{2}^{+}$.

**Fig. 2:**
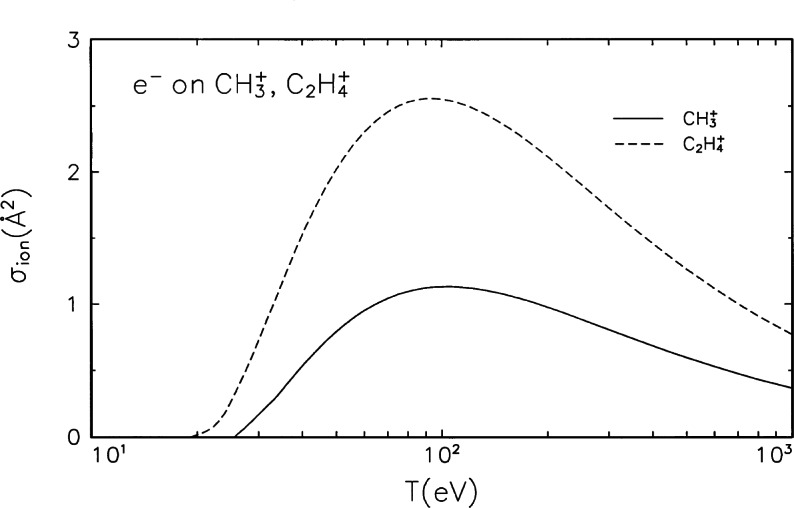
Electron-impact ionization cross sections for ${\mathrm{CH}}_{3}^{+}$ and $C_{2}H_{4}^{+}$.

**Fig. 3:**
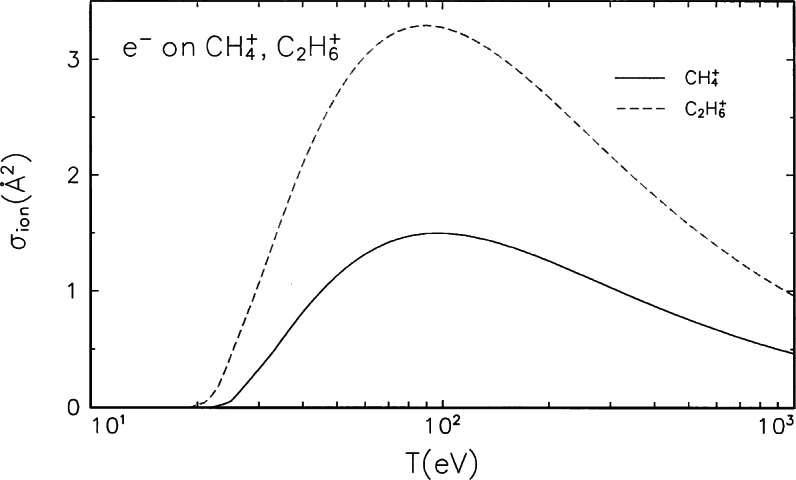
Electron-impact ionization cross section for ${\mathrm{CH}}_{4}^{+}$ and $C_{2}H_{6}^{+}$.

**Fig. 4:**
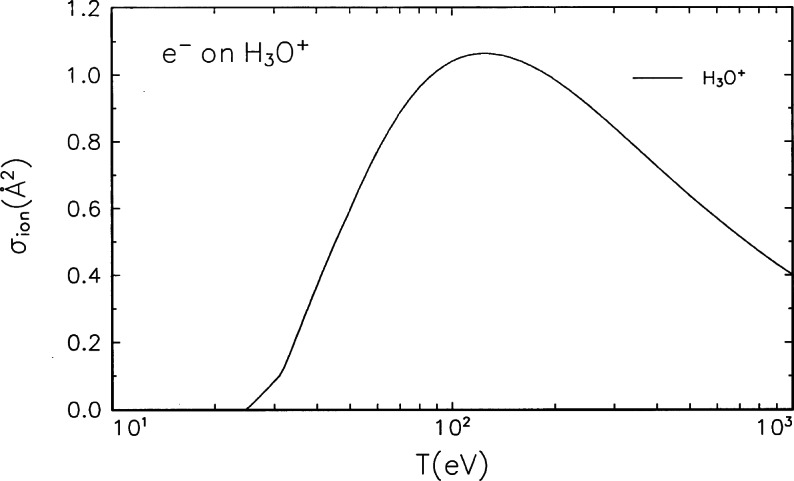
Electron-impact ionization cross section for H_3_O^+^.

We present the BEB cross sections from Eq. (2) for ${\mathrm{CH}}_{2}^{+}$, ${\mathrm{CH}}_{3}^{+}$, ${\mathrm{CH}}_{4}^{+}$, $C_{2}H_{2}^{+}$, $C_{2}H_{4}^{+}$, $C_{2}H_{6}^{+}$, and H_3_O^+^ in Figs. 1–4. The molecular constants *B*, *U*, and *N* for the molecules are listed in Table 1. For all molecular ions except ${\mathrm{CH}}_{4}^{+}$, molecular geometries were computed using a hybrid density functional (B3LYP) [10,11] with 6-31G(d) basis sets. For ${\mathrm{CH}}_{4}^{+}$, B3LYP/6-31G(d) gave an incorrect molecular symmetry (C_2_ point group instead of C_2_*_v_*), so the geometry was computed using frozen-core, second-order perturbation (MP2) theory with 6-31G(d) basis sets. The B3LYP or MP2 geometries were used for all subsequent calculations of *B* and *U*. Kinetic energies *U* for all orbitals, and binding energies *B* for the inner orbitals, were calculated at the Hartree-Fock (HF) level using 6-311G(d,p) basis sets. More accurate, correlated values of *B* were obtained for the outer-valence orbitals by using frozen-core Green’s function (OVGF) methods [12,13] and 6-311+G(d,p) basis sets. For the important threshold ionization, *B* values were obtained by using frozen-core coupled cluster theory [CCSD(T)], with the single and double excitation operators included iteratively [14] and the contribution from connected triples estimated perturbatively [15]. Dunning’s correlation-consistent valence-triple-zeta (cc-pVTZ) basis sets [16] were used for the CCSD(T) calculations. The HF calculations were performed using the GAMESS [17] program package; all other calculations employed Gaussian 98^1^ [18].

In general, when an electron collides with a molecular ion we get

$$e^{-}+{\mathrm{AB}}^{+}\to {A+B}^{+}+e^{-},$$ (3)

$$\text{or}\;A^{+}+{B+e}^{-}.$$ (4)

$$e^{-}+{AB}^{+}\to {AB}^{++}+2e^{-},$$ (5)

$$\text{or}\;A^{+}+B^{+}+2e^{-},$$ (6)

$$\text{or}\;A^{++}+{B+2e}^{-},$$ (7)

$$\text{or}\;{A+B}^{++}+2e^{-}.$$ (8)

Processes (3) and (4) are dissociation without ionization, while processes (5) through (8) are the ionizing events described by the BEB model. The model calculates the sum of all processes (5) through (8) that lead to the ejection of a bound electron. Moreover, the model also assumes—erroneously—that all energy transfers from the incident electron to the target molecule that exceed the ionization energy of a given molecular orbital result in ionization. This is an assumption common to all binary-encounter type theories. Although such an assumption may be valid for atoms, molecules may dissociate without ionizing even if energy transfers exceed the orbital binding energies. If processes (3) and (4) are significant for energy transfers above orbital binding energies, then the BEB model will overestimate ionization cross sections. More discussions on this point can be found in Ref. [5].

Experimentally, the production of doubly charged ions can be detected directly when the doubly charged ions have reasonably long lifetimes. In reality, most doubly charged molecular ions quickly dissociate into two singly charged fragments, making it almost impossible to distinguish processes (3) and (4) from the dissociation of doubly charged ions by Coulomb repulsion, i.e., process (6). For this reason, it is difficult to distinguish processes (3) and (4) from process (6) simply by detecting singly charged ions unless coincidence measurements of all products are performed. The usual experimental procedure is to measure the cross section for producing any ion, i.e., (3) through (8). Then, processes (3) and (4) are measured separately, and subtracted from the total ion production cross section. This subtraction introduces large uncertainties in the resulting experimental ionization cross sections.

The direct measurement of only the doubly charged ions—processes (5), (7), and (8)—tends to produce small cross sections compared to the total ionization cross section because of the high probability for the rapid break-up of the doubly charged ions as shown in Ref. [7] for CO^+^. As another example, Bahati et al. [8] recently reported experimental cross sections for the process of

$$e^{-}+H_{3}O^{+}\to H_{3}O^{++}+2e^{-}$$ (9)

Their measurement corresponds to process (5) only, and their peak cross section is 0.049 Å^2^ at *T* ≈ 125 eV. The position of the peak is in agreement with the BEB cross section in Fig. 4, but the magnitude is almost 1/20 of the BEB cross section. This discrepancy and the similar discrepancy in CO^+^ (experiment is lower by a factor of 1/12 at the peak) measured by the same group [19] is a strong indication that either dissociative ionization is the dominant process, or the break-up of the doubly charged ions is faster than the experimental capability to detect.

## Figures and Tables

**Table 1:** Molecular point group, molecular orbitals (MO), electron binding energy *B*, kinetic energy *U*, and electron occupation number *N* for ${\mathrm{CH}}_{2}^{+}$, ${\mathrm{CH}}_{3}^{+}$, ${\mathrm{CH}}_{4}^{+}$, $C_{2}H_{2}^{+}$, $C_{2}H_{4}^{+}$, $C_{2}H_{6}^{+}$, and H_3_O^+^

Molecule (point group)	MO	*B*(eV)	*U*(eV)	*N*
${\mathrm{CH}}_{2}^{+}$ (C_2_*_v_*)	1*a*_1_	319.07	436.58	2
	2*a*_1_	33.16	38.98	2
	1*b*_2_	27.04	29.90	2
	3*a*_1_	22.17	37.74	1
${\mathrm{CH}}_{3}^{+}$ (D_3_*_h_*)	$1a_{1}^{\prime }$	317.93	436.46	2
	$2a_{1}^{\prime }$	33.55	37.57	2
	1*e*′	25.59	29.56	4
${\mathrm{CH}}_{4}^{+}$ (C_2_*_v_*)	1*a*_1_	315.92	436.20	2
	2*a*_1_	33.67	33.40	2
	3*a*_1_	25.87	27.79	2
	1*b*_2_	24.48	28.73	2
	1*b*_1_	22.08	29.42	1
$C_{2}H_{2}^{+}$ (D_∞_*_h_*)	1*σ*_g_	316.56	435.87	2
	1*σ*_u_	316.48	436.63	2
	2*σ*_g_	34.59	49.00	2
	2*σ*_u_	27.96	35.66	2
	3*σ*_g_	26.06	34.23	2
	1*σ*_u_	21.00	31.42	3
$C_{2}H_{4}^{+}$ (D_2_)	1*a*	315.15	436.08	2
	1*b*_1_	315.12	436.44	2
	2*a*	33.52	40.73	2
	2*b*_1_	28.22	36.01	2
	1*b*_2_	24.41	27.06	2
	3*a*	23.30	35.07	2
	1*b*_3_	21.81	29.99	2
	2*b*_3_	19.21	29.54	1
$C_{2}H_{6}^{+}$ (D_3_*_d_*)	1*a*_1g_	314.28	436.27	2
	1*a*_2u_	314.28	436.30	2
	2*a*_1g_	31.76	33.19	2
	2*a*_2u_	29.16	38.10	2
	1*e*_u_	23.07	26.29	4
	1*e*_g_	21.38	30.07	4
	3*a*_1g_	19.04	30.07	1
H_3_O^+^ (C_3_*_v_*)	1*a*_1_	571.51	794.48	2
	2*a*_1_	45.59	72.42	2
	1*e*	29.90	52.11	4
	3*a*_1_	24.7^a^	65.41	2

a Experimental value from Ref. [8].

## Footnotes

1 Certain commercial equipment, instruments, or materials are identified in this paper to foster understanding. Such identification does not imply recommendation or endorsement by the National Institute of Standards and Technology, nor does it imply that the materials or equipment identified are necessarily the best available for the purpose.
